# Application of Patient-Generated Health Data Among Older Adults With Cancer: Scoping Review

**DOI:** 10.2196/57379

**Published:** 2025-02-04

**Authors:** Yesol Kim, Geonah Kim, Hyeonmi Cho, Yeonju Kim, Mona Choi

**Affiliations:** 1 College of Nursing and Brain Korea 21 FOUR Project Yonsei University Seoul Republic of Korea; 2 College of Nursing Gyeongsang National University Jinju Republic of Korea; 3 Severance Hospital Yonsei University Health System Seoul Republic of Korea; 4 Mo-Im Kim Nursing Research Institute College of Nursing Yonsei University Seoul Republic of Korea; 5 Research Institute of AI and Nursing Science College of Nursing Gachon University Incheon Republic of Korea; 6 Yonsei Evidence Based Nursing Centre, A JBI Affiliated Group Seoul Republic of Korea

**Keywords:** patient-generated health data, wearable devices, patient-reported outcomes, patient-centered care, older adults, cancer, scoping review

## Abstract

**Background:**

The advancement of information and communication technologies has spurred a growing interest in and increased applications of patient-generated health data (PGHD). In particular, PGHD may be promising for older adults with cancer who have increased survival rates and experience a variety of symptoms.

**Objective:**

This scoping review aimed to identify the characteristics of research on PGHD as applied to older adults with cancer and to assess the current use of PGHD.

**Methods:**

Guided by Arksey and O’Malley as well as the JBI (Joanna Briggs Institute) methodology for scoping reviews, 6 electronic databases were searched: PubMed, Embase, CINAHL, Cochrane Library, Scopus, and Web of Science. In addition, the reference lists of the selected studies were screened to identify gray literature. The researchers independently screened the literature according to the predefined eligibility criteria. Data from the selected studies were extracted, capturing study, participant, and PGHD characteristics.

**Results:**

Of the 1090 identified studies, 88 were selected. The publication trend gradually increased, with a majority of studies published since 2017 (69/88, 78%). Almost half of the studies were conducted in North America (38/88, 43%), followed by Europe (30/88, 34%). The most common setting in which the studies were conducted was the participant’s home (69/88, 78%). The treatment status varied; the median sample size was 50 (IQR 33.8-84.0). The devices that were used to measure the PGHD were classified as research-grade wearable devices (57/113, 50.4%), consumer-grade wearable devices (28/113, 24.8%), or smartphones or tablet PCs for mobile apps (23/113, 20.4%). More than half of the studies measured physical activity (69/123, 56.1%), followed by patient-reported outcomes (23/123, 18.7%), vital signs (13/123, 10.6%), and sleep (12/123, 9.8%). The PGHD were mainly collected passively (63/88, 72%), and active collection methods were used from 2015 onward (20/88, 23%). In this review, the stages of PGHD use were classified as follows: (1) identification, monitoring, review, and analysis (88/88, 100%); (2) feedback and reporting (32/88, 39%); (3) motivation (30/88, 34%); and (4) education and coaching (19/88, 22%).

**Conclusions:**

This scoping review provides a comprehensive summary of the overall characteristics and use stages of PGHD in older adults with various types and stages of cancer. Future research should emphasize the use of PGHD, which interacts with patients to provide patient-centered care through patient engagement. By enhancing symptom monitoring, enabling timely interventions, and promoting patient involvement, PGHD have the potential to improve the well-being of older adults with cancer, contributing to better health management and quality of life. Therefore, our findings may provide valuable insights into PGHD that health care providers and researchers can use for geriatric cancer care.

**Trial Registration:**

Open Science Framework Registry OSF.IO/FZRD5; https://doi.org/10.17605/OSF.IO/FZRD5

## Introduction

### Background

Advancements in early detection and treatment modalities have led to a consistent increase in the survival rates of patients diagnosed with cancer. In the United States, the number of survivors of cancer reached 18.1 million in 2022, and this figure is projected to increase to 26 million by 2040 [1]. With rising cancer survival rates, there is a notable trend of aging among survivors of cancer. In 2022, over two-thirds (67%) of US survivors of cancer were aged 65 years or older [1].

Older adults often experience multiple chronic health conditions, and survivors of cancer show an even higher prevalence of these issues compared to those without cancer [2]. Common co-occurring conditions in older patients with cancer include musculoskeletal conditions, such as osteoarthritis and sciatica; mental health disorders, such as depression and anxiety; and cardiovascular diseases, such as hypertension and coronary artery disease [2,3]. These conditions may exacerbate susceptibility to functional decline, with older survivors of cancer facing an accelerated deterioration in grip strength, gait speed, and overall physical performance compared to their peers without cancer [4]. Moreover, the presence of multiple chronic conditions and functional limitations in older survivors of cancer are associated with a reduced quality of life, increased risk of early mortality, and significant economic burden [5,6].

Given the challenges associated with both cancer and aging, innovative approaches to monitoring and managing the health of older survivors of cancer are essential for improving their health and well-being outcomes. The collection and sharing of pertinent health data can offer survivors of cancer and their health care providers an opportunity to enhance their health [7]. The rise of consumer technologies, such as smartphone apps and wearable devices, has paved the way for the creation and use of tools that enable individuals to gather their own health-related data [8]. These data are related to their physical behavior (eg, step count or sleep patterns), physiological parameters (eg, heart rate or blood pressure), biochemical markers (eg, blood glucose levels), and environmental factors (eg, air quality or temperature) in which they are present.

With the advancement of information and communication technologies, the interest in and the applications of patient-generated health data (PGHD) are growing. PGHD refer to “health-related data—including health history, symptoms, biometric data, treatment history, lifestyle choices, and other information—created, recorded, gathered, or inferred by or from patients or their designees (ie, care partners or those who assist them)” [9]. PGHD are distinct from data sourced from clinical environments or interactions with health care providers. The primary distinction is that PGHD are recorded by patients and not providers, and their sharing is directed by the patients [9]. Patients can collect these data either passively or actively through mobile health apps, wearable devices (eg, fitness bands), medical devices (eg, blood glucose monitors), or survey instruments on mobile devices [7]. Passive data collection occurs when data are gathered automatically with minimal patient input, typically using automated systems or sensors. By contrast, active data collection requires patients to be actively involved, typically by responding to surveys.

PGHD enable the consistent, long-term monitoring of diverse health-related information that can be easily exchanged between patients and health care providers [10]. These data can complement the information that health care systems typically gather through providers, offering individualized baseline indicators. With PGHD, both patients and health care providers can quickly discern changes in the health status, allowing for more appropriate and prompt interventions [11].

PGHD have found applications in monitoring and managing various long-term health conditions. For instance, PGHD can be used to identify undiagnosed obesity and diabetes, assess the risk of these conditions, and predict glycemic events in patients with diabetes [12]. In the context of HIV care, PGHD help alleviate financial and temporal pressures by reducing the need for frequent in-person consultations [11]. Continuous monitoring through PGHD enhances medication adherence and increases patients’ understanding of their health status [11]. For survivors of cancer, who face higher posttreatment health risks, PGHD enable real-time tracking of health parameters such as activity levels, symptom progression, and medication adherence, facilitating early detection of issues and more personalized interventions [7]. Beyond individual care, PGHD can also benefit larger survivors of cancer communities by contributing to population-level insights, informing public health strategies, enhancing survivorship guidelines, and supporting research on cancer survivorship.

While there is the potential for using PGHD in older adults with cancer, few review studies have focused on PGHD in these patients. A comprehensive understanding of the characteristics and use of PGHD in older adults with cancer will help improve patient engagement in health care and enable researchers to apply PGHD in geriatric cancer care. This will ultimately lead to the effective application of PGHD in enhancing the health and well-being of older adults with cancer.

### Objectives

This scoping review aimed to identify the characteristics of research on PGHD as applied to older adults with cancer and to assess the current use of PGHD. The main research question for the scoping review was “What is the present landscape of research concerning PGHD in older adults with cancer?”

The specific research questions were as follows: (1) What are the characteristics of the studies conducted on PGHD in older adults with cancer? (2) What are the characteristics of older adults with cancer who have been the subject of PGHD-related research? and (3) What are the characteristics of PGHD that have been gathered from older adults with cancer?

## Methods

### Design

This scoping review was conducted following the guidelines of Arksey and O’Malley [13] and the JBI (Joanna Briggs Institute) methodology for scoping reviews [14]. This scoping review was also performed according to the PRISMA-ScR (Preferred Reporting Items for Systematic Reviews and Meta-Analyses Extension for Scoping Review) guidelines Multimedia Appendix 1 [15]. The population, concept, and context for this scoping review were as follows: (1) population—older adults with cancer, (2) concept—PGHD, which were collected using devices; and (3) context—no limitation for setting. The protocol of the scoping review was registered in the Open Science Framework Registry.

### Search Strategy

In this study, 6 electronic databases—PubMed, Embase, CINAHL, Cochrane Library, Scopus, and Web of Science—were searched for relevant literature. Considering the population, concept, and context of this scoping review, the search strategy was developed by combining keywords and index terms from each database (Multimedia Appendix 2). PGHD as a concept was introduced as a Medical Subject Headings (MeSH) term by PubMed in 2018. To retrieve relevant literature before the introduction of PGHD as a MeSH term, we further constructed PGHD-specific search terms by combining the keywords “patient-generated” and “devices.” In addition, the search was limited to the index terms included in the titles and abstracts of the studies to ensure proper identification of the literature. An experienced librarian at the researcher’s university confirmed this search strategy. A literature search was conducted using a confirmed search strategy on October 21, 2022. After the literature search, the reference lists of the selected studies were screened to identify the gray literature.

### Eligibility Criteria and Study Selection

To assess the eligibility of studies on PGHD in older adults with cancer, the inclusion criteria were as follows: (1) studies that included older adults with a mean or median age of 65 years or older; (2) studies that included older adults with cancer, regardless of the type of cancer or treatment status; and (3) studies that applied PGHD, including wearable devices that measure patients’ physical activity or physiological features or mobile phones that measure patient-reported outcomes (PROs). The exclusion criteria were as follows: (1) studies in which PGHD were selectively used in some of the participants; (2) studies comparing results using PGHD for validation of other tools; (3) publications other than original studies, such as editorials, letters, or protocols; (4) publications that were not peer reviewed, such as abstracts, conference proceedings, dissertations, or theses; and (5) studies written in languages other than English or Korean.

The studies identified in the literature search were exported to the reference management software program (EndNote X9; Clarivate Analytics). After deleting duplicate results using EndNote, the results were extracted with a Microsoft Excel program (Microsoft Corp) for literature screening. Two researchers (Yesol K and GK) independently selected studies based on the inclusion and exclusion criteria. The titles and abstracts were initially reviewed; subsequently, the full texts were reviewed to select studies for final inclusion. If the two researchers did not agree on a particular study, a consensus was reached through discussion and the participation of a third researcher (MC).

### Data Extraction

Data from the selected studies were extracted using Microsoft Excel in a structured data charting format, which was constructed by the researchers. It comprised the study, participant, and PGHD characteristics. First, the study characteristics included published year, country, continent, study design, and dataset. Second, the participant characteristics included the study setting, type of cancer, treatment status, and sample size. Lastly, the PGHD characteristics included the type of device that was used to measure PGHD, type of assessment by PGHD, on-body locations of the wearable devices that measure PGHD, duration of wear of the devices that measure PGHD, type of PGHD collection, and type of PGHD use. The types of PGHD collection were categorized as passive (using sensor-based devices that automatically acquire and transfer PGHD from patients), active (requiring active patient engagement to collect PGHD that are not easily collected automatically), and combined methods [16]. The types of PGHD use were categorized into the following four categories: (1) identification, monitoring, review, and analysis; (2) feedback and reporting; (3) motivation; and (4) education and coaching [16]. Two researchers (Yesol K and GK) independently extracted the data, and disagreements, if any, were resolved by consensus through discussion and the participation of a third researcher (MC).

### Data Analysis

The data extracted from this scoping review are summarized and presented using descriptive statistics. The results were mapped and are presented in the form of tables, charts, or figures according to the purpose and research questions of this scoping review.

## Results

### Study Selection

**Figure 1:**
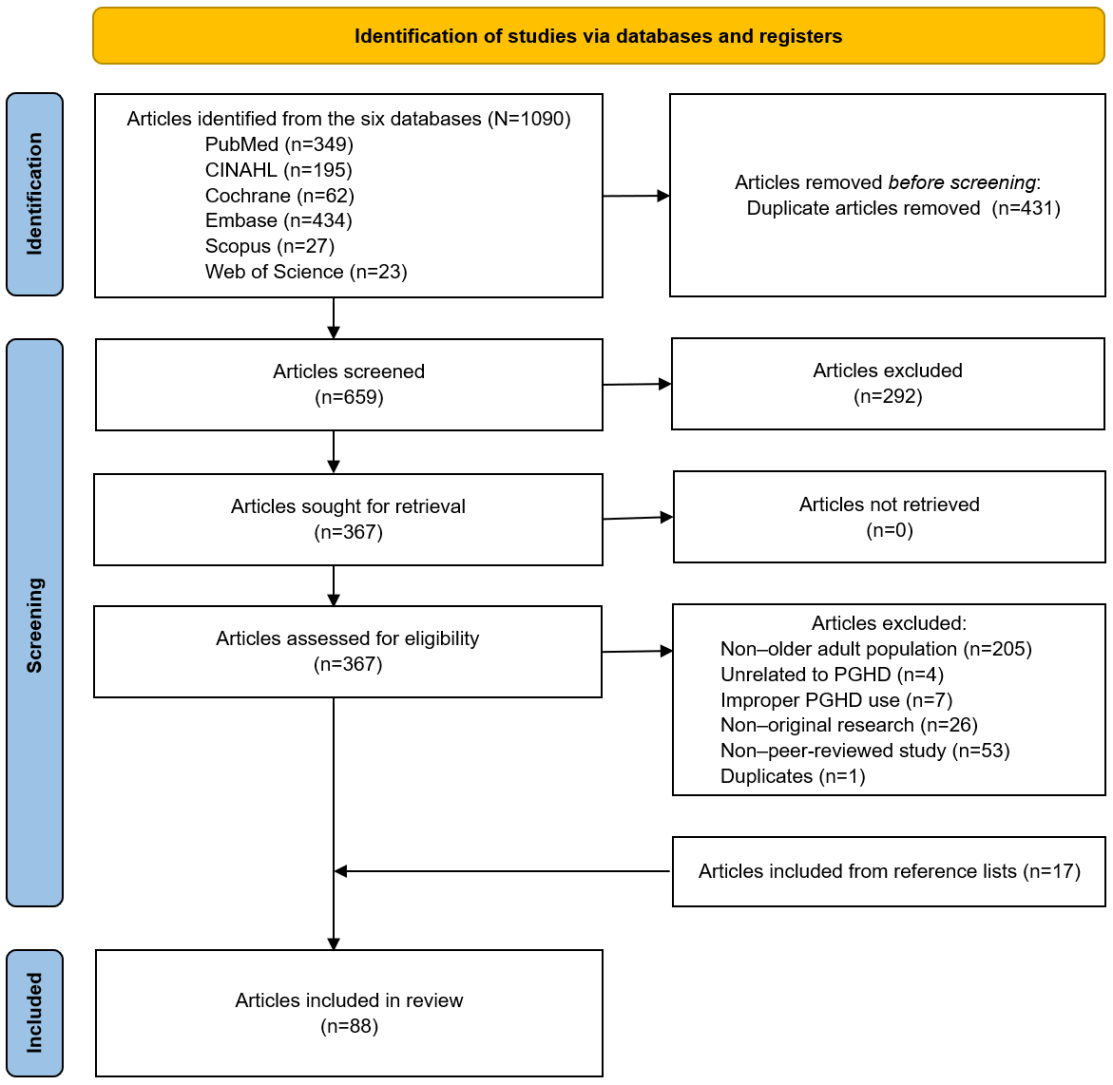
PRISMA flow diagram of this study. PGHD: patient-generated health data; PRISMA: Preferred Reporting Items for Systematic Reviews and Meta-Analyses.

The literature selection process is illustrated in Figure 1 as a PRISMA (Preferred Reporting Items for Systematic Reviews and Meta-Analyses) flow diagram. A total of 1090 articles were retrieved from 6 database searches. After the elimination of duplicates, the titles and abstracts of 659 (60.5%) articles were reviewed. Subsequently, the full texts of 367 (33.7%) articles were reviewed to determine eligibility for this review. The reasons for the exclusion of articles from the full-text review are listed in Figure 1. After the screening, 71 (6.5%) articles were selected. A review of the references of the 71 selected articles led to the additional selection of 17 articles, resulting in a selection of a total of 88 articles for the review [17-104].

### Study Characteristics

The studies included in this scoping review were published between 2006 and 2022 (Multimedia Appendix 3). There has been a discernible upward trend in publication volume, with more than 70% of the articles published since 2017 (69/88, 78%) [36-104]. Geographically, North America emerged as the leading contributor to the body of research, accounting for 43% (38/88) of the studies. The study designs were categorized into nonexperimental and experimental designs. Among studies with nonexperimental designs, a survey-based study design was most frequently adopted (39/88, 44%). Among studies with experimental designs, a pre-experimental design was most frequently adopted (28/88, 32%). Data derived from original research constituted the basis for more than 60% of the studies (59/88, 67%; Table 1).

**Table 1:** Characteristics of included studies (n=88).

Study characteristics			Studies, n (%)	Reference numbers
**Continents**				
	Asia		8 (9)	[37,48,53,76,82,97,99,102]
	Europe		30 (34)	[17,18,20,28,32,33,35,38,40,42,43,45,50,58,61,66,69,71,73,74,78,79,83-85,89,90,96,101,104]
	North America		38 (43)	[21-23,29,30,36,41,44,46,47,49,51,54-57,60,62-65,67,68,70,75,77,80,81,86,87,91-95,98,100,103]
	South America		1 (1)	[52]
	Africa		0 (0)	N/A^a^
	Oceania		11 (13)	[19,24-27,31,34,39,59,72,88]
**Study design**				
	**Nonexperimental design**			
		Survey	39 (44)	[17-19,21-23,26-28,30,34,35,37-39,41,44,46-49,51,55,57,59,62,67,68,73,74,76,78,81,83,89,95,97,99,102]
		Qualitative study	3 (3)	[33,42,96]
		Methodological research	2 (2)	[91,94]
	**Experimental design**			
		True experimental design	13 (15)	[25,31,50,56,60,70,82,84,86-88,93,98]
		Quasi-experimental design	3 (3)	[43,58,66]
		Pre-experimental design	28 (32)	[20,24,29,32,36,40,45,52-54,61,63-65,69,71,72,75,77,79,80,85,90,92,100,101,103,104]
**Dataset**				
	Original research		59 (67)	[17,18,20,24,26-32,37,39,41,43,44,46-50,52-54,57,60,61,63,64,66-70,73-75,77,79-87,90-93,96-103]
	Other research		24 (27)	[21,25,33-36,38,40,42,45,51,56,58,59,65,71,72,76,78,88,89,94,95,104]
	National data or cohort data		5 (6)	[19,22,23,55,62]

^a^N/A: not applicable.

### Participant Characteristics

The participant characteristics are presented in Table 2. Most studies were conducted at the participants’ homes (69/88, 78%). Among the included studies, none focused solely on older adults living alone. Only 15 (17%) of the 88 studies distinguished participant characteristics based on whether they lived alone or with others, and most participants in these studies lived with others. Of the 88 studies, 64 (73%) specified the selection criteria for study participants according to cancer type. These studies included 76 cases of various types of cancer, the most common of which was prostate cancer (22/76, 29%). Regarding gender proportions, 66 (75%) of the 88 studies had a higher proportion of men, while 20 (23%) studies had a higher proportion of women. Of these, 18 (20%) studies focused exclusively on men, all involving patients with prostate cancer. In contrast, 3 (3%) studies focused exclusively on women, all involving patients with breast cancer. Half of the studies were conducted on patients who received active treatment (29/88, 33%) or surgery (15/88, 17%); a quarter were conducted on survivors of cancer (22/88, 25%). In this review, survivors of cancer are defined as individuals who have completed their primary cancer treatments—such as surgery, chemotherapy, or radiation therapy—and are in the posttreatment phase. The median sample size was 50 (IQR 33.8-84.0), and more than 40% of the studies included fewer than 50 (39/88, 44%) participants. In the studies included in this review, the mean age of participants ranged from 65 (SD 7) to 76.9 (SD 10.5) years.

**Table 2:** Characteristics of participants (n=88).

Participant characteristics		Studies, n (%)	Reference numbers
**Setting**			
	Home	69 (78)	[18-27,30-35,37-52,54-65,67,69-72,74,76,78-80,82-84,86-90,92,93,95-97,100,104]
	Hospital (outpatient)	3 (3)	[29,36,53]
	Hospital (inpatient)	5 (6)	[17,62,73,99,102]
	Home and hospital (combined)	11 (13)	[28,68,75,77,81,85,91,94,98,101,103]
**Type of cancer^a^**			
	Prostate	22 (29)	[21-23,25,31,32,34,38,40,42,43,50,51,54,56,60,61,70,82,85,88,92]
	Gastrointestinal	21 (28)	[20,23,28,33,35,38,41,45,50,58,64-66,68,71,72,77,78,91,94,101]
	Lung	18 (24)	[20,26,27,30,36,37,46,48,49,53,57,75-77,86,94,97,101]
	Bladder, urethra, or urothelial	5 (7)	[63,66,83,92,98]
	Others	10 (13)	[19,23,59,66,72,79,80,84,100,103]
**Gender proportions**			
	Higher proportion of men	66 (75)	[18,20-22,25-32,34-44,46-48,50,51,53-56,58-61,63-66,68-71,73,74,76-79,82-85,88-92,95-99,101,102]
	Higher proportion of women	20 (23)	[17,19,23,24,33,49,52,57,62,67,72,75,80,81,86,87,93,94,100,104]
	Equal proportion of men and women	1 (1)	[103]
	Not reported	1 (1)	[45]
**Treatment status**			
	Diagnosed with cancer with no limitation of treatment	11 (13)	[26,27,29,44,48,53,59,62,76,79,88]
	Received active treatment	29 (33)	[21,28,30,32,33,36,37,39,40,42,43,45,51,52,54,56,64,65,67,78,80,82-85,92,97,103,104]
	Underwent surgery	15 (17)	[58,63,66,71,74,75,77,80,81,89-91,94,98,101]
	Survivor of cancer	22 (25)	[19,20,22-25,31,34,35,38,41,49,50,55,60,61,72,87,93,95,96,100]
	Received end-of-life and palliative care	4 (5)	[47,73,99,102]
	Not reported	7 (8)	[17,18,46,57,69,70,86]
**Sample size (participants)**			
	≤49	39 (44)	[21,24,28-30,32,33,39,41,42,45,46,52-54,63,68,71-73,76,77,79,82,83,85,86,88,90,93,95-100,102-104]
	50-99	28 (32)	[17,18,20,26,27,37,40,47,48,56-60,64,65,69,74,75,78,80,81,87,89,91,92,94,101]
	100-149	11 (13)	[22,23,25,34,43,44,55,61,66,67,84]
	≥150	10 (11)	[19,31,35,36,38,49-51,62,70]

^a^There were a total number of 76 cases of various types of cancer among 64 studies.

### PGHD Characteristics

The characteristics of the PGHD identified in each study are summarized in Table 3. In this review, research-grade wearable devices were used in more than half of the cases (57/113, 50.4%): these included the ActivPal Monitor (PAL Technologies Ltd), Stepwatch Activity Monitor (Modus Health), and ActiGraph GT3x Activity Monitor. Consumer-grade wearable devices were used in 28 (24.8%) cases: these included Fitbit, Apple Watch, Garmin band, Microsoft Band, Samsung Gear, and Xiaomi MiBand. Smartphones, tablet PCs, and computers were used to collect data using mobile apps or web-based surveys in 25 (22.1%) cases. Other devices, including smart mattresses and an instant camera, were used in 3 (2.7%) cases.

**Table 3:** Characteristics of patient-generated health data (n=88).

Patient-generated health data characteristics			Studies, n (%)	Reference numbers			
**Device type^a^**							
	Research-grade wearable devices	57 (50.4)		[17-28,30,31,34,35,37-39,41,47-51,54-56,59-67,72,73,76,78,82,87,88,93,95,101]			
	Consumer-grade wearable devices	28 (24.8)		[45,46,57,60,68,70,72,74,77,80,86,87,89-91,93-95,97,98,100,103]			
	Smartphones, tablet PCs, or computers for mobile apps or surveys	25 (22.1)		[29,32,33,36,40,42-44,52,53,58,69-71,75,79,81,83-85,89,91,92,96,104]			
	Other devices	3 (2.7)		[99,100,102]			
**Assessment type^b^**							
	Physical activity	69 (56.1)		[17-28,31,34,35,38,39,41,45,46,48-50,52,54-57,59-68,70,72,74,76,77,80-82,86-91,93-95,98,100-103]			
	Sleep	12 (9.8)		[17,18,30,37,47,48,51,57,73,80,91,103]			
	Vital signs	13 (10.6)		[28,45,63,78,89-91,97,99]			
	Patient-reported outcomes	23 (18.7)		[29,32,33,36,40,42-44,53,58,69-71,75,79,83-85,89,91,92,96,104]			
	Others	6 (4.9)		[45,63,78,90,91,100]			
**On-body location of wearable device^c^**							
	Wrist	35 (29.9)		[17,18,21,28,30,37,41,45,47,48,51,54,57,61-63,72-74,76-78,80,86,87,89-91,93,94,97,100,103]			
	Waist	11 (9.4)		[26,27,49,60,67,68,70,82,93,95]			
	Hip	13 (11.1)		[19,22,23,31,34,38,39,50,52,59,65,72,88]			
	Lower extremity (leg, ankle, or foot)	8 (6.8)		[20,35,66,67,87,95,101]			
	Others (neck, chest, arm, finger, skin, or forehead)	13 (11.1)		[18,28,45,55,63,66,78,90,95]			
	Nonbody (cloth or mattress)	7 (6.0)		[45,46,60,63,98,99,102]			
	Not admitted (mobile apps or camera)	30 (25.6)		[24,25,29,32,33,36,40-44,53,56,58,64,69-71,75,79,81,83-85,89,91,92,96,100,104]			
**Wearing period of device^a^**							
	≤1 week	47 (41.6)		[17-23,26,29,34-39,41,44,46-49,52,53,55,57,59,62,63,66,67,73,76,78,82,87,95,97,100]			
	>1 week or ≤4 weeks (1 month)	23 (20.4)		[27,28,30,32,50,51,54,61,64,65,68,72,75,88-90,93,94,96]			
	5-12 weeks (3 months)	25 (22.1)		[24,31,40,42,43,45,54,60,74,77,79,85,86,89-93,98,101,103]			
	>12 weeks (3 months)	16 (14.2)		[25,33,56,58,69-72,80,81,83,84,87,99,102,104]			
	Not reported	2 (1.8)		[18,70]			
**Collection** **type**							
	Passive collection	63 (72)		[17-28,30,31,34,35,37-39,41,45-52,54-57,59-68,72-74,76-78,80-82,86-88,93-95,97-99,101-103]			
	Active collection	20 (23)		[29,32,33,36,40,42-44,53,58,69,71,75,79,83-85,92,96,104]			
	Combined collection	5 (6)		[38,70,89,91,100]			
**Collection support by health care providers**		20 (23)		[32,36,40,42-44,47,58,74,83,86,87,89,90,92,97,98,101,103,104]			

^a^The total number is 113 due to multiple categories.

^b^The total number is 123 due to multiple categories.

^c^The total number is 117 due to multiple categories.

Regarding the type of assessment by PGHD, more than half of the assessments (69/123, 56.1%) focused on physical activity measured through step count, moderate-to-vigorous physical activity, and sedentary behavior. Sleep, including the sleep-wake cycle, circadian rhythm, and movement in bed, were measured in 12 (9.8%) cases. There were 23 (18.7%) cases of PROs, including symptoms and quality of life, and 6 (4.9%) cases of other assessments, such as body weight and exposure to light intensity.

Regarding the on-body locations of wearable devices that measure PGHD, approximately one-third were worn on the wrist (35/116, 30.2%). Additionally, mobile apps or cameras were used without attaching them to the body in 25.9% (30/116) of cases. Regarding the wearing period of the device, the device was worn for ≤1 week in 47 (41.6%) cases and for >12 weeks (3 months) in 16 (14.2%) cases.

**Figure 2:**
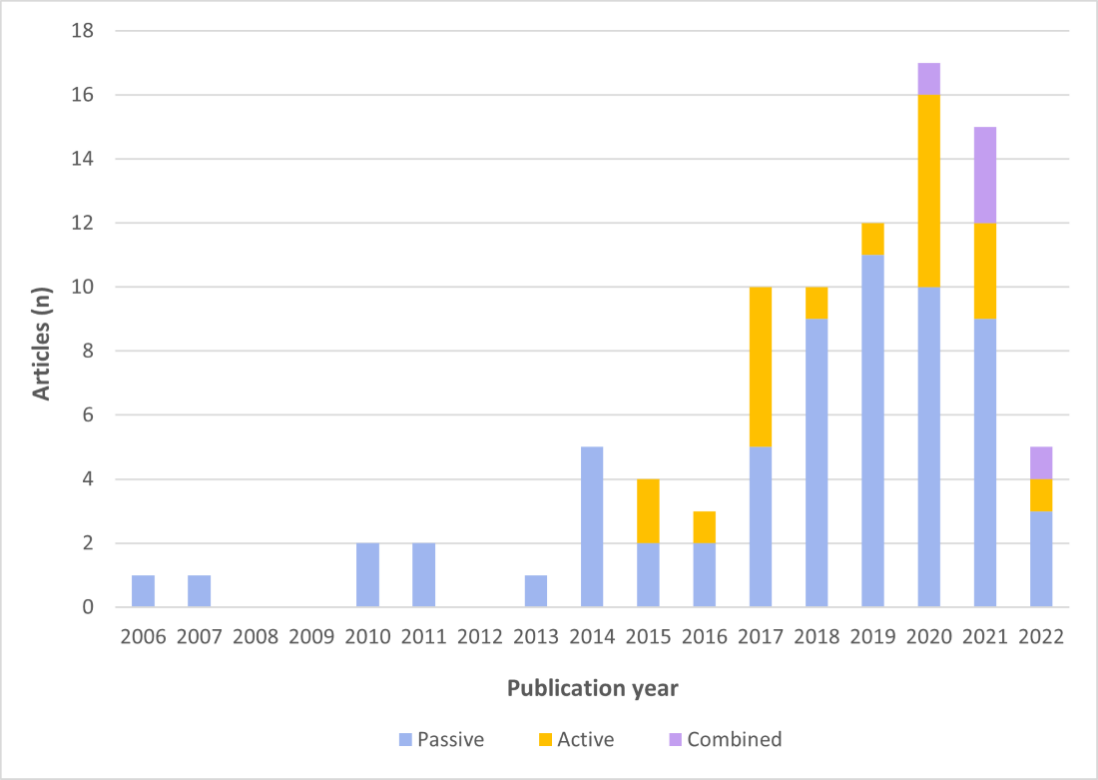
Type of patient-generated health data collection by year.

PGHD were collected in various ways, including passive, active, and combined collection (Table 3 and Figure 2). PGHD were primarily passively collected from participants (63/88, 72%), constituting most of the included studies published between 2006 and 2022. Furthermore, in 20 (22%) studies, PGHD were actively collected from participants from 2015 onward. However, few studies combined passive and active collection methods for PGHD (5/88, 6%), which have been used in research since 2020.

In detail, 25 studies actively collected PGHD, including combined collection methods. Among these, smartphones, tablet PCs, or computers were the predominant devices for active data collection through mobile apps or surveys (23/25, 92%). Except for 1 study that used computers, of the 22 studies using smartphones and tablet PCs, 6 (27%) used the Android (Google LLC) operating system, 5 (23%) used iOS (Apple Inc), 3 (14%) used both, and 8 (36%) did not specify the operating system. Regarding device ownership, 10 (45%) studies provided devices to participants, 3 (14%) studies relied on participants’ own devices, 4 (18%) studies used both approaches, and 5 (23%) studies did not report on this aspect.

Among the studies included in this review, approximately a quarter (21/88, 24%) had a sample size of over 100 participants. The PGHD monitoring period varied from 1 day to 24 weeks, with over 60% (13/21) of studies reporting a duration of 1 week or less. Additionally, more than three-quarters of these studies (16/21) collected PGHD passively, primarily using wearables, including both research-grade and consumer-grade devices. Of the 88 studies, 67 (76%) reported on participants’ adherence to PGHD collection, with adherence rates ranging from 36% to 100%. More than half of these studies (36/67, 54%) reported an adherence rate above 80%. In addition, the dropout rates reported in 57 longitudinal studies ranged from 0% to 72.4%. Approximately one-fifth of these studies (13/57, 23%) completed PGHD collection with full participant retention.

In this review, health care providers supported PGHD collection from participants in only 20 (23%) studies. Health care providers assisted with the synchronization of the devices, troubleshooting of technical issues, and so forth (10/20, 50%) [42,44,74,86,87,89,98,101,103,104]. They also provided reminders to participants, encouraging PGHD collection (9/20, 45%) [32,36,40,43,58,83,92,97,101]. In addition, health care providers directly assisted with data collection (1/20, 5%) [47] and requested additional information if participants did not respond appropriately (1/20, 5%) [90]. In the case of support methods, phone calls (11/20, 55%) [40,42,43,47,83,86,87,89,90,92,97], text messages (1/20, 5%) [86], app push notifications (4/20, 20%) [32,40,43,58], emails (1/20, 5%) [83], and in-person contact (2/20, 10%) [44,47] were identified.

In this study, the stages of PGHD use were classified as follows: (1) identification, monitoring, review, and analysis; (2) feedback and reporting; (3) motivation; and (4) education and coaching (Multimedia Appendix 4). Identification, monitoring, review, and analysis of PGHD were implemented across all studies (88/88, 100%). Other stages were implemented by interacting with the participants and health care providers. Feedback and reporting were applied in 34 (39%) studies by using PGHD to aid participants in checking their data and receiving technical advice [24,26,32,33,38,40,42,43,52-54,56,58,60,63,69-72,74,75, 79,82,83,86-90,92,93,98,101,104]. PGHD was used as a motivator for participants in approximately one-third of the studies (30/88, 34%) [24,32,33,40,42,43,53,54,56,58,60,63,65, 70,71,74,75,77,79,82,86-88,92,93,96,98,100,101,103]. It included directly encouraging participants to generate data, reminding them, setting goals, and supporting them in the process of generating PGHD. Approximately one-fifth of the studies (19/88, 22%) provided education and coaching using PGHD, enhancing the attainment of additional information, knowledge, and skills [25,32,40,53,56,58,70-72,75,77,79,82,87, 88,92,93,100,104].

## Discussion

### Principal Findings

This scoping review presents a broad overview and summary of the studies on PGHD use among older adults with cancer. Following are the key findings of this scoping review, which included a total of 88 studies published between 2006 and 2022. A variety of devices were used to collect PGHD, including research-grade wearable devices, consumer-grade wearable devices, and smartphones or tablet PCs. These devices assessed different PGHD in older adults with cancer, including physical activity, sleep, vital signs, and PROs. In most studies, PGHD were collected passively, requiring no additional effort from the patient, other than wearing the device. Active and mixed PGHD collection methods have recently been applied to research. In most studies, there was no support from health care providers when PGHD were collected from older adults with cancer. PGHD were used for the identification, monitoring, review, and analysis in all studies included in the review. However, fewer than half of the studies identified advanced use of PGHD, such as feedback and reporting, motivation, and education and coaching.

### Study Characteristics

Since 2017, an increasing number of studies have been published on the applications of PGHD in older adults with cancer. This may be attributed to the fact that the MeSH term for PGHD was introduced in PubMed in 2018. Given the current publication trend, this can be considered an early stage of use of PGHD-related research in older adults with cancer, indicating that there is a need for further research. Approximately one-third of the included studies used secondary sources for PGHD; most were original, while 5 used cohort data. These 5 studies used the following cohort data: the Baltimore Longitudinal Study of Aging [55] and the National Health and Nutrition Examination Survey [19,22,23,62] from the United States, which measured the participants’ physical activity. The advantage of using data from a cohort is that PGHD can be collected from a relatively large number of people and can be used by many researchers simultaneously, which can have a positive impact on patient health care. Therefore, it is recommended to consider including PGHD when constructing a large cohort study.

### Participants Characteristics

Approximately 80% (69/88) of the studies reviewed in this analysis focused on the application of PGHD in home settings, underscoring its well-established use outside of hospital environments. Our findings indicate that it is valuable to provide health care providers with continuously collected data on health status at home through PGHD [105]. PGHD can offer patients valuable insights into their health by allowing them to monitor their health at home between hospital visits or during self-care [106]. Moreover, the application of PGHD eliminates time and location constraints, thereby enhancing patient accessibility and participation in their health care [107].

In this review, the cancer treatment statuses of the participants differed. Notably, the cancer survivorship phase, which constituted a quarter of this review, often lacked ongoing health care beyond periodic hospital follow-up visits. Multiple studies have highlighted the unmet need of survivors of cancer for continued care postactive treatment [108,109]. However, PGHD offers several benefits for survivors of cancer. Petersen [7] indicated that it is beneficial for survivors of cancer to apply for PGHD to improve and promote their health. For instance, the collection of PGHD at home, encompassing various health aspects, such as symptoms, can assist health care providers in identifying and managing health issues effectively [7]. In other words, PGHD have the potential to improve patient engagement among survivors who do not frequently receive hospital care and allow health care providers to access patients in a clinical setting. This makes it a promising tool for enhancing the quality of life and health outcomes of survivors of cancer, thereby encouraging future studies to actively use PGHD in their management.

### PGHD Characteristics

We found that wearable devices were used to collect approximately three-quarters of the PGHD. These wearable devices have expanded the scope of research beyond the confines of electronic health records by enabling integrated data acquisition from patients [110]. Previous reviews reporting on the use of wearable devices across various health domains indicated that approximately 86% data were collected using consumer-grade wearable devices, including Fitbits, whereas approximately 14% were from research-grade devices [111]. In contrast, in this study, research-grade wearable devices were used in approximately half of the cases and consumer-grade in approximately a quarter. This difference can be attributed to the perceived suitability and higher measurement accuracy of research-grade wearable devices in research settings, particularly among older adults with cancer, which is the target population of this review [112]. Despite this, recent clinical studies have shown an increase in the use of consumer-grade wearable devices, and their reliability and validity have been established [113]. Given their relative affordability and lower burden of wear, consumer-grade wearable devices, which enable continuous usage, are considered to have a high potential for widespread use [114]. Therefore, rather than distinguishing between the types of wearable devices, the focus should be on the selection of the most appropriate device for collecting PGHD, considering the specific circumstances and environments of older adults with cancer.

In this review, more than half of the collected PGHD items focused on physical activity. Previous studies have shown that older adults with cancer tend to engage in less physical activity compared to middle-aged patients [115]. Moreover, physical activity has been demonstrated to enhance physical function and overall quality of life in older adults with cancer [116,117]. This underscores the importance of prioritizing increased physical activity as a critical factor in improving the health of this population. PGHD can increase health awareness and patient engagement, leading to positive effects on physical activity [107]. Essentially, it can help older adults with cancer in making health behavior changes that enhance physical activity while supporting their autonomy [7]. Moreover, self-monitoring of physical activity using PGHD can motivate individuals to engage in these activities [118]. Therefore, future research using PGHD to monitor and encourage physical activity among older adults with cancer may improve their health outcomes.

This review identified PGHD that measure PROs, including symptoms, in older adults with cancer, which is crucial for enhancing treatment and health outcomes [119]. While visiting a hospital in person and objectively measuring their condition is challenging for patients with cancer, self-reporting conditions, including symptoms, can be more beneficial [120]. Patients were able to report the symptoms they experienced in real time from their homes through PGHD, and contrary to expectations, they did not find it burdensome to report daily [40]. PGHD offer a means for patients with cancer to communicate their condition directly to health care providers, thereby eliminating time and distance constraints and benefiting both the patients and providers [7]. Especially, older patients with cancer are at a higher risk of reporting more severe symptoms [121]. Therefore, enabling older patients with cancer to assess their condition, including symptoms, through PGHD without time and distance limitations can facilitate the early detection of deterioration and personalized care [119]. However, none of the studies included in this review actively involved older adults with cancer in selecting the PGHD to be collected. Therefore, future studies should consider selecting and collecting PGHD items based on participants’ needs to enhance engagement.

In addition, based on the data collected through the PROs, reports or alerts about symptoms were provided to patients, and health care providers offered advice or interventions for symptom management [32,42,79]. This indicates that patients, even when not in the hospital, can experience gratitude and psychological comfort, knowing that they are connected to and receiving care from health care providers and are managing their health issues [40]. PGHD enabled patients to gain confidence in self-management of their symptoms, suggesting a positive impact on their health management.

This review categorizes PGHD collection into passive, active, and combined methods, with the passive collection being predominantly used (63/88, 72%). This differs from a prior review of PGHD for disease prevention and health promotion, in which active or partially active methods were more common in healthy populations [16]. Passive methods that use wearable devices for objective data collection without an added burden have been in use since 2006. Given the focus on older adults with cancer in this review, simpler passive methods have been widely adopted. In addition, health care providers tend to view passively collected PGHD as more reliable and more challenging to manipulate, as indicated in previous studies [122].

In contrast, approximately one-fifth of the studies used the active collection method, which showed a gradual increase over time. This method, which involves direct and active participation of the patients in data collection, allows patients to report their subjective states, contributing to enhanced patient engagement [123]. However, a previous review identified conflicting characteristics between passive and active collection [16]. Passive approaches were found to be less burdensome for patients, but may reduce their participation, whereas active approaches require voluntary effort, but motivate patient involvement [16]. Considering these characteristics, a combined collection approach may be ideal for PGHD. In this review, only approximately 6% (5/88) of the studies used a combined collection method, which started to appear in 2020. For example, in the study by Low et al [91], passive data collection using wearable and smartphone sensors for PGHD, combined with active data collection of patient-reported symptoms, facilitated personalized and adaptive symptom assessments to enable early detection of adverse events. This approach may ultimately enhance patient well-being by enabling timely interventions throughout long-term cancer treatment and recovery. Thus, future research should consider using a combined data collection approach to comprehensively assess the health status of older adults with cancer.

An adequate level of digital literacy is essential for patients to generate PGHD, comprehend their significance, and receive motivation for behavioral changes to enhance adherence. However, concerns have arisen about the potential disparities among those unable to collect or use PGHD for various reasons. Age is a contributing factor, as older adults may struggle to adopt new technologies owing to their unfamiliarity [124]. Moreover, patients with cancer often experience higher physical and psychological symptom burdens than do those without cancer, potentially reducing their inclination to use digital health technologies [125]. Therefore, future studies may require additional strategies to identify and enhance digital literacy among older adults with cancer. A tailored approach that integrates demographic factors (eg, age or gender), geographic factors (eg, place of residence), socioeconomic factors (eg, education level), behavioral factors (eg, information-seeking behavior), and social factors (eg, social support) could promote more effective use of wearable devices and mobile apps for PGHD collection and use [126].

Regarding the PGHD use stages, all studies used PGHD at the first stage, primarily collecting and analyzing PGHD, to identify and monitor the health status of older adults with cancer for research purposes. This aligns with a previous review of disease prevention and health promotion, in which many studies reviewed and analyzed PGHD [16]. However, the first stage of PGHD use can be considered as merely using the gathered information for analysis. To facilitate bidirectional rather than unidirectional communication through PGHD, patients must be able to check or access their generated data. The studies included in this review initially identified and analyzed PGHD, and then PGHD were used for advanced use depending on the purpose of the study. In only approximately one-third of the studies, the patients were provided with information and feedback on PGHD to help them understand their condition; PGHD motivated patients to achieve health-related goals and was used for education or coaching interventions. Therefore, offering actionable recommendations based on PGHD is expected to increase patient engagement in health behaviors, especially among older adults. This implies that the use of PGHD should not be limited to the generation of data through wearable devices but should extend to accessing this data and receiving feedback that motivates behavior change. Similarly, when collecting PROs including symptoms via mobile apps, a bidirectional structure is necessary in which patients receive advice or guidance in addition to reporting their information.

To promote the widespread use of PGHD in research involving older adults with cancer, it is crucial to increase adherence to digital devices for PGHD measurements. This necessitates identifying the needs, barriers, and facilitators for older adults with cancer regarding PGHD, and providing education where there are gaps to encourage adherence. In particular, patients with lower digital literacy encounter greater barriers to using PGHD [16,127]; this can negatively impact the collection and use of PGHD, emphasizing the need for initiatives to improve digital literacy.

Cultural considerations are also essential for adopting PGHD among older adults with cancer. A survey conducted across four European countries revealed that over half of the participants recognized the usefulness of PGHD and expressed willingness to share it with health care providers to improve their own health [128]. In contrast, for community-dwelling older adults in urban areas of South Korea, family or peer influence was reported as both a facilitator and a barrier to adopting digital technologies [129]. Considering the unique cultural attitudes and behavioral intentions that influence technology adoption is crucial for effectively collecting and using PGHD among older adults with cancer [130].

Moreover, the quality of PGHD is a significant concern, influenced by user-related factors, device and technology, and data governance [110]. Researchers should not only focus on collecting PGHD but also on enhancing data quality for effective use. Additionally, the establishment of standards or frameworks for assessing the quality, reliability, and accuracy of PGHD from various devices is essential. Meeting these requirements will enable the sharing and integration of PGHD with health care providers for use in patient care and health management in clinical settings. Ultimately, future research should aim to integrate PGHD from nonhospital settings into health care systems, such as electronic health records or research registries, to ensure continuity of care.

### Limitations

This scoping review endeavored to apply a rigorous methodology; however, it has a few limitations. First, the PubMed MeSH term for PGHD has only been included since 2018, indicating that some studies, corresponding to PGHD, may not have been searched. To compensate for this, search terms corresponding to PGHD such as wearable devices and patient report were included. Furthermore, even if PGHD were not explicitly stated, studies deemed to be PGHD through discussions among researchers were included in the review. Additionally, the references of the selected studies were reviewed to ensure that there were no omissions. Second, this review did not include gray literature in the literature selection process. By excluding theses and conference papers, and focusing only on peer-reviewed studies, it is possible that recent literature containing the latest information on PGHD was excluded. Third, this study included older patients with various types of cancer at different treatment stages, suggesting a need for caution regarding the heterogeneity related to cancer in the application of PGHD. Therefore, careful interpretation of the results of this review is warranted, and future research should focus on understanding the use of PGHD according to the specific types of cancer and treatment stages. Finally, although the inclusion criteria of this review focused on studies with a mean or median age of 65 years or older, some studies may have included participants younger than 65 years. This could affect the generalizability of the findings to the geriatric population, as the presence of younger participants may not fully reflect the health characteristics and challenges faced by older adults.

### Conclusions

This scoping review investigated and summarized the overall characteristics and use stages of PGHD among older adults with various types and stages of cancer. Research involving PGHD in older adults with cancer is gradually increasing and is primarily conducted at home through wearable devices or mobile apps. PGHD encompass a collection of physiological variables, such as physical activity, sleep, and vital signs, along with PROs, using both passive and active methods, and have been used in various aspects of research. However, while PGHD collected from older adults with cancer were universally used for analysis within the studies, their use for providing feedback, motivation, and interventions that could impact patients and researchers was limited. These findings suggest that future research should emphasize the use of PGHD, which interact with patients to provide patient-centered care through patient engagement. Furthermore, health care, especially in areas such as physical activity and symptom management, is critical for older adults with cancer, and PGHD can positively influence their active participation in health care. Therefore, our findings may provide insights into PGHD that can be used by health care providers and researchers in future research in the context of geriatric cancer care.

## Data Availability

The protocol of the scoping review was registered in the Open Science Framework Registry. All data generated in the review is available in the main text and in Multimedia Appendix 3 amd Multimedia Appendix 4.

## Appendix

Multimedia Appendix 1 PRISMA-ScR (Preferred Reporting Items for Systematic Reviews and Meta-Analyses Extension for Scoping Reviews) checklist.

Multimedia Appendix 2 Search strategies for PubMed, EMBASE, and CINAHL.

Multimedia Appendix 3 Proportion of studies that focused on various stages of patient-generated health data use (n=88).

Multimedia Appendix 4 Proportion of studies that focused on various stages of patient-generated health data use (n=88).

## Abbreviations

JBI: Joanna Briggs Institute
MeSH: Medical Subject Headings
PGHD: patient-generated health data
PRISMA: Preferred Reporting Items for Systematic Reviews and Meta-Analyses
PRISMA-ScR: Preferred Reporting Items for Systematic Reviews and Meta-Analyses Extension for Scoping Review
PRO: patient-reported outcome
