# Supplementary material for: Application of Patient-Generated Health Data Among Older Adults With Cancer: Scoping Review
Source: J Med Internet Res. 2025 Feb 4;27:e57379. doi: 10.2196/57379 (PMC11836591; doi:10.2196/57379)
Supplement: Multimedia Appendix 2 [file jmir_v27i1e57379_app2.docx]

(a) PubMed

| **Search number** | **Search terms** | **Search results** |
| --- | --- | --- |
| 1 | elder*[Title/Abstract] OR senior*[Title/Abstract] OR "older adult*"[Title/Abstract] OR "older individual*"[Title/Abstract] OR "older person*"[Title/Abstract] OR "older people"[Title/Abstract] OR "older men"[Title/Abstract] OR "older women"[Title/Abstract] OR "older patient*"[Title/Abstract] OR "old age"[Title/Abstract] | 544,162 |
| 2 | "Aged"[Mesh] | 3,419,341 |
| 3 | #1 OR #2 | 3,601,743 |
| 4 | cancer*[Title/Abstract] OR tumor*[Title/Abstract] OR tumour*[Title/Abstract] OR neoplas*[Title/Abstract] OR malignan*[Title/Abstract] OR carcinom*[Title/Abstract] OR oncolog*[Title/Abstract] | 3,890,613 |
| 5 | "Neoplasms"[Mesh] | 3,746,239 |
| 6 | #4 OR #5 | 4,917,304 |
| 7 | #3 AND #6 | 1,002,323 |
| 8 | "patient-generated health data"[Title/Abstract] OR "patient-generated data"[Title/Abstract] OR "person-generated health data"[Title/Abstract] OR "person-generated data"[Title/Abstract] | 412 |
| 9 | "Patient Generated Health Data"[Mesh] | 110 |
| 10 | #8 OR #9 | 480 |
| 11 | "patient-generated"[Title/Abstract] OR "person-generated"[Title/Abstract] OR "patient-reported"[Title/Abstract] OR "self-report*"[Title/Abstract] OR "self-monitor*"[Title/Abstract] OR "self-rated"[Title/Abstract] | 264,362 |
| 12 | "Self Report"[Mesh] OR "Patient Reported Outcome Measures"[Mesh] OR "Monitoring, Physiologic"[Mesh] | 244,956 |
| 13 | #11 OR #12 | 462,736 |
| 14 | wearable*[Title/Abstract] OR "fitness tracker*"[Title/Abstract] OR "fitness tracking"[Title/Abstract] OR "activity tracker*"[Title/Abstract] OR "activity tracking"[Title/Abstract] OR "activity monitor*"[Title/Abstract] OR "step count*"[Title/Abstract] OR accelerometr*[Title/Abstract] OR actigraph*[Title/Abstract] OR "fitness band*"[Title/Abstract] OR smartband*[Title/Abstract] OR wristband*[Title/Abstract] OR armband*[Title/Abstract] OR smartwatch*[Title/Abstract] OR fitbit[Title/Abstract] OR garmin[Title/Abstract] OR xiaomi[Title/Abstract] OR huawei[Title/Abstract] OR withings[Title/Abstract] OR "mobile phone*"[Title/Abstract] OR smartphone*[Title/Abstract] OR "mobile app"[Title/Abstract] OR "mobile apps"[Title/Abstract] OR "mobile application*"[Title/Abstract] OR "smartphone app"[Title/Abstract] OR "smartphone apps"[Title/Abstract] OR "smartphone application*"[Title/Abstract] OR "tablet computer*"[Title/Abstract] OR "tablet PC"[Title/Abstract] | 79,252 |
| 15 | "Wearable Electronic Devices"[Mesh] OR "Fitness Trackers"[Mesh] OR "Accelerometry"[Mesh] OR "Actigraphy"[Mesh] OR "Cell Phone"[Mesh] OR "Smartphone"[Mesh] OR "Mobile Applications"[Mesh] OR "Computers, Handheld"[Mesh] | 58,507 |
| 16 | #14 OR #15 | 107,395 |
| 17 | #13 AND #16 | 20,617 |
| 18 | #10 OR #17 | 20,930 |
| 19 | #7 AND #18 | 349 |

(b) EMBASE

| **Search number** | **Search terms** | **Search results** |
| --- | --- | --- |
| 1 | elder*:ab,ti OR senior*:ab,ti OR 'older adult*':ab,ti OR 'older individual*':ab,ti OR 'older person*':ab,ti OR 'older people':ab,ti OR 'older men':ab,ti OR 'older women':ab,ti OR 'older patient*':ab,ti OR 'old age':ab,ti | 737,332 |
| 2 | 'aged'/exp | 3,588,251 |
| 3 | #1 OR #2 | 3,843,392 |
| 4 | cancer*:ti,ab OR tumor*:ti,ab OR tumour*:ti,ab OR neoplas*:ti,ab OR malignan*:ti,ab OR carcinom*:ti,ab OR oncolog*:ti,ab | 5,254,993 |
| 5 | 'malignant neoplasm'/exp | 4,035,767 |
| 6 | #4 OR #5 | 6,154,789 |
| 7 | #3 AND #6 | 1,057,394 |
| 8 | 'patient-generated health data':ti,ab OR 'patient-generated data':ti,ab OR 'person-generated health data':ti,ab OR 'person-generated data':ti,ab | 367 |
| 9 | 'patient-generated':ti,ab OR 'person-generated':ti,ab OR 'patient-reported':ti,ab OR 'self-report*':ti,ab OR 'self-monitor*':ti,ab OR 'self-rated':ti,ab | 360,242 |
| 10 | self report'/exp OR 'patient-reported outcome'/exp OR 'patient monitoring'/exp | 442,275 |
| 11 | #9 OR #10 | 647,632 |
| 12 | wearable*:ti,ab OR 'fitness tracker*':ti,ab OR 'fitness tracking':ti,ab OR 'activity tracker*':ti,ab OR 'activity tracking':ti,ab OR 'activity monitor*':ti,ab OR 'step count*':ti,ab OR accelerometr*:ti,ab OR actigraph*:ti,ab OR 'fitness band*':ti,ab OR smartband*:ti,ab OR wristband*:ti,ab OR armband*:ti,ab OR smartwatch*:ti,ab OR fitbit:ti,ab OR garmin:ti,ab OR xiaomi:ti,ab OR huawei:ti,ab OR withings:ti,ab OR 'mobile phone*':ti,ab OR smartphone*:ti,ab OR 'mobile app':ti,ab OR 'mobile apps':ti,ab OR 'mobile application*':ti,ab OR 'smartphone app':ti,ab OR 'smartphone apps':ti,ab OR 'smartphone application*':ti,ab OR 'tablet computer*':ti,ab OR 'tablet pc':ti,ab | 95,517 |
| 13 | wearable computer'/exp OR 'wearable sensor'/exp OR 'activity tracker'/exp OR 'smart watch'/exp OR 'accelerometry'/exp OR 'actimetry'/exp OR 'accelerometer'/exp OR 'mobile phone'/exp OR 'smartphone'/exp OR 'mobile application'/exp OR 'tablet computer'/exp | 97,032 |
| 14 | #12 OR #13 | 136,279 |
| 15 | #11 AND #14 | 21,475 |
| 16 | #8 OR #15 | 21,696 |
| 17 | #7 AND #16 | 434 |

(c) CINAHL

| **Search number** | **Search terms** | **Search results** |
| --- | --- | --- |
| S1 | TI ( elder* OR senior* OR "older adult*" OR "older individual*" OR "older person*" OR "older people" OR "older men" OR "older women" OR "older patient*" OR "old age" ) OR AB ( elder* OR senior* OR "older adult*" OR "older individual*" OR "older person*" OR "older people" OR "older men" OR "older women" OR "older patient*" OR "old age" ) | 261,055 |
| S2 | (MH "Aged+") | 934,162 |
| S3 | S1 OR S2 | 1,019,840 |
| S4 | TI ( cancer* OR tumor* OR tumour* OR neoplas* OR malignan* OR carcinom* OR oncolog* ) OR AB ( cancer* OR tumor* OR tumour* OR neoplas* OR malignan* OR carcinom* OR oncolog* ) | 695,208 |
| S5 | (MH "Neoplasms+") | 645,528 |
| S6 | S4 OR S5 | 878,812 |
| S7 | S3 AND S6 | 186,196 |
| S8 | TI ( "patient-generated health data" OR "patient-generated data" OR "person-generated health data" OR "person-generated data" ) OR AB ( "patient-generated health data" OR "patient-generated data" OR "person-generated health data" OR "person-generated data" ) | 175 |
| S9 | TI ( "patient-generated" OR "person-generated" OR "patient-reported" OR "self-report*" OR "self-monitor*" OR "self-rated" ) OR AB ( "patient-generated" OR "person-generated" OR "patient-reported" OR "self-report*" OR "self-monitor*" OR "self-rated" ) | 196,721 |
| S10 | (MH "Self Report+") OR (MH "Patient-Reported Outcomes+") OR (MH "Monitoring, Physiologic+") | 130,226 |
| S11 | S9 OR S10 | 283,004 |
| S12 | TI ( wearable* OR "fitness tracker*" OR "fitness tracking" OR "activity tracker*" OR "activity tracking" OR "activity monitor*" OR "step count*" OR accelerometr* OR actigraph* OR "fitness band*" OR smartband* OR wristband* OR armband* OR smartwatch* OR fitbit OR garmin OR xiaomi OR huawei OR withings OR "mobile phone*" OR smartphone* OR "mobile app" OR "mobile apps" OR "mobile application*" OR "smartphone app" OR "smartphone apps" OR "smartphone application*" OR "tablet computer*" OR "tablet PC" ) OR AB ( wearable* OR "fitness tracker*" OR "fitness tracking" OR "activity tracker*" OR "activity tracking" OR "activity monitor*" OR "step count*" OR accelerometr* OR actigraph* OR "fitness band*" OR smartband* OR wristband* OR armband* OR smartwatch* OR fitbit OR garmin OR xiaomi OR huawei OR withings OR "mobile phone*" OR smartphone* OR "mobile app" OR "mobile apps" OR "mobile application*" OR "smartphone app" OR "smartphone apps" OR "smartphone application*" OR "tablet computer*" OR "tablet PC" ) | 29,362 |
| S13 | (MH "Wearable Sensors+") OR (MH "Fitness Trackers") OR (MH "Accelerometry+") OR (MH "Actigraphy") OR (MH "Cellular Phone") OR (MH "Smartphone") OR (MH "Mobile Applications") OR (MH "Computers, Portable+") | 33,918 |
| S14 | S12 OR S13 | 50,196 |
| S15 | S11 AND S14 | 9,071 |
| S16 | S8 OR S15 | 9,201 |
| S17 | S7 AND S16 | 195 |
