# Supplementary figures and images for: Application of Patient-Generated Health Data Among Older Adults With Cancer: Scoping Review
Source: J Med Internet Res. 2025 Feb 4;27:e57379. doi: 10.2196/57379 (PMC11836591; doi:10.2196/57379)

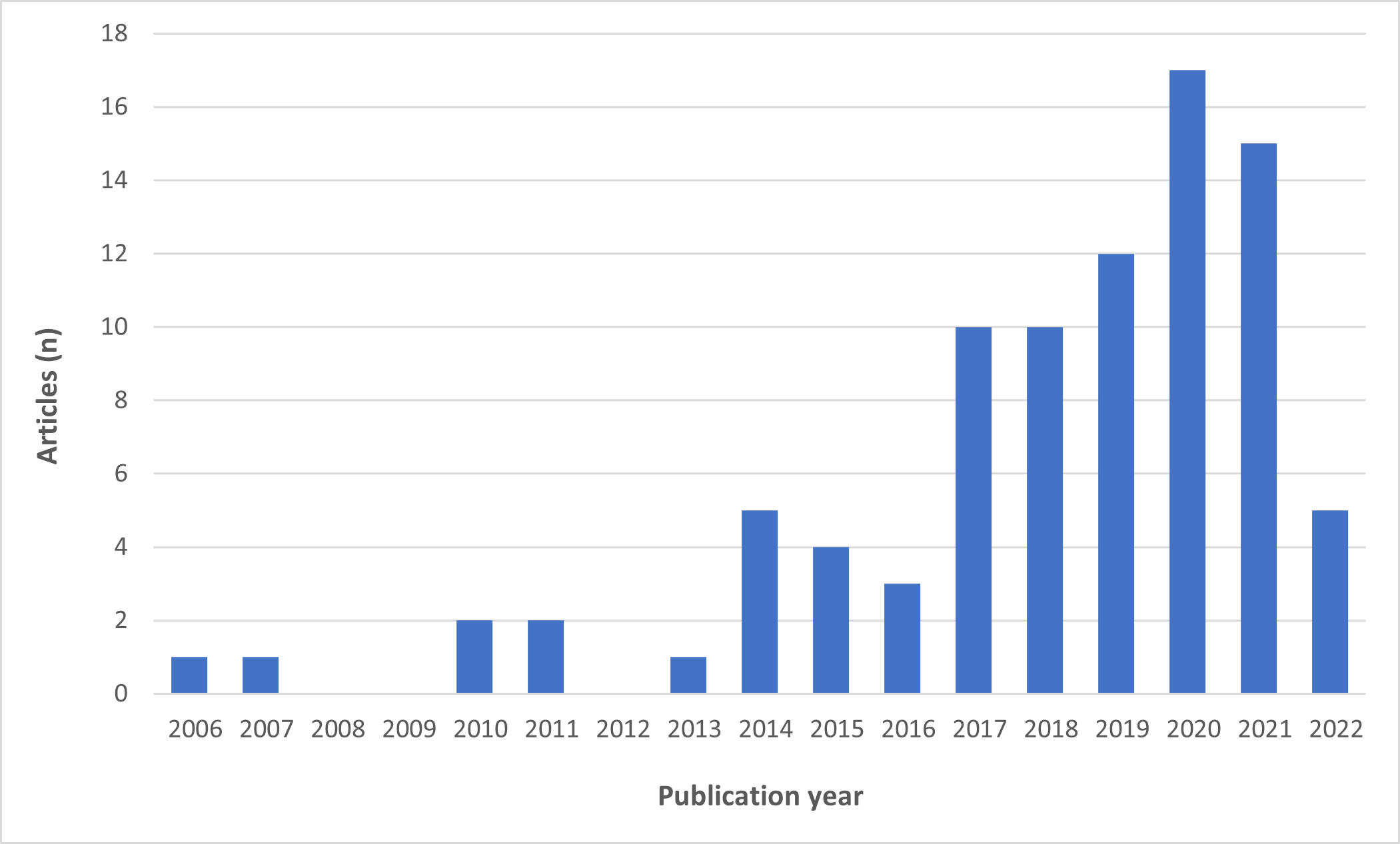

Supplement: Multimedia Appendix 3 [file jmir_v27i1e57379_app3.png]

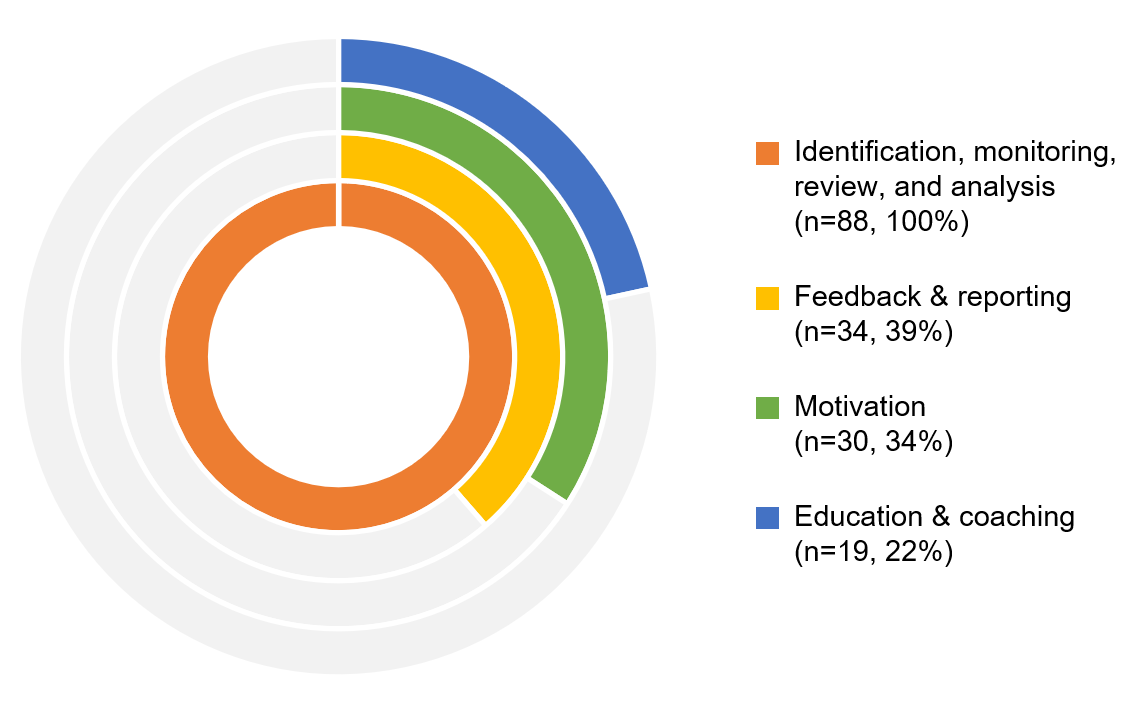

Supplement: Multimedia Appendix 4 [file jmir_v27i1e57379_app4.png]
